# Determining the contribution of a high-fructose corn syrup formulation to hepatic glycogen synthesis during ad-libitum feeding in mice

**DOI:** 10.1038/s41598-020-69820-3

**Published:** 2020-07-30

**Authors:** Giada DiNunzio, Getachew D. Belew, Alejandra N. Torres, João Gabriel Silva, Luis P. Silva, Cristina Barosa, Ludgero Tavares, John G. Jones

**Affiliations:** Center for Neurosciences and Cell Biology, University of Coimbra, UC-Biotech, Biocant Park, 3060-197 Cantanhede, Portugal

**Keywords:** Biochemistry, Chemical biology

## Abstract

Excessive sugar intake including high-fructose corn syrup (HFCS) is implicated in the rise of obesity, insulin resistance and non-alcoholic fatty liver disease. Liver glycogen synthesis is influenced by both fructose and insulin signaling. Therefore, the effect of HFCS on hepatic glycogenesis was evaluated in mice feeding ad-libitum. Using deuterated water: the fraction of glycogen derived from triose-P sources, Krebs cycle substrates, and direct pathway + cycling, was measured in 9 normal-chow fed mice (NC) and 12 mice fed normal chow plus a 55% fructose/45% glucose mix in the drinking water at 30% w/v (HFCS-55). This was enriched with [U-^13^C]fructose or [U-^13^C]glucose to determine the contribution of each to glycogenesis. For NC, direct pathway + cycling, Krebs cycle, and triose-P sources accounted for 66 ± 0.7%, 23 ± 0.8% and 11 ± 0.4% of glycogen synthesis, respectively. HFCS-55 mice had similar direct pathway + cycling (64 ± 1%) but lower Krebs cycle (12 ± 1%, *p* < 0.001) and higher triose-P contributions (24 ± 1%, *p* < 0.001). HFCS-55-fructose contributed 17 ± 1% via triose-P and 2 ± 0% via Krebs cycle. HFCS-55-glucose contributed 16 ± 3% via direct pathway and 1 ± 0% via Krebs cycle. In conclusion, HFCS-55 supplementation resulted in similar hepatic glycogen deposition rates. Indirect pathway contributions shifted from Krebs cycle to Triose-P sources reflecting HFCS-55-fructose utilization, while HFCS-55-glucose was incorporated almost exclusively by the direct pathway.

## Introduction

**Figure 1:**
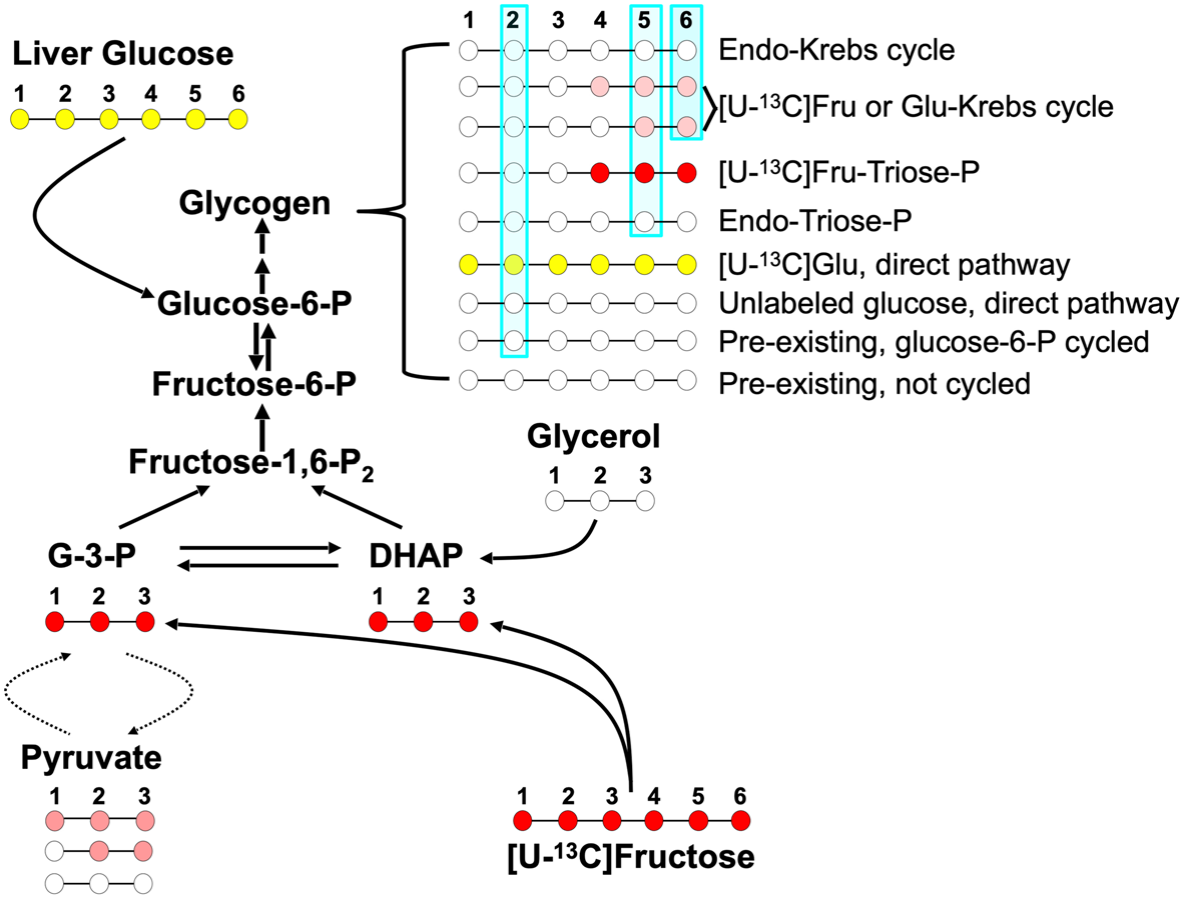
Schematic of glycogen synthesis from exogenous [U-^13^C]fructose, unlabeled endogenous sources and hepatic glucose enriched with exogenous [U-^13^C]glucose. ^13^C-isotopomers of triose-P intermediates and glycogen originating from [U-^13^C]fructose are shown by red circles while glycogen ^13^C-isotopomers that originated via the Krebs cycle are shown as pink. Glycogen formed from hepatic glucose via the direct pathway is shown as yellow. Unlabeled glycogen and unlabeled precursors are represented by unfilled circles. For simplicity, some metabolic intermediates were omitted and only those ^13^C-isotopomers within the 456-triose moiety of glycogen are shown. The blue shading represents glycogen molecules that are enriched with ^2^H in positions 2, 5 and 6_*s*_ from ^2^H_2_O.

Increased intake of refined sugar, either as sucrose or as high-fructose corn syrup (HFCS), is implicated in the surge of obesity and related diseases such as non-alcoholic fatty liver disease (NAFLD) and Type 2 Diabetes (T2D). The most common form of HFCS, HFCS-55, consists of 55% fructose and 45% glucose with the same level of sweetness as sucrose. To better understand the mechanisms by which excessive sugar intake promotes the development of NAFLD and T2D, there have been extensive studies with rodent models provided with diets high in refined sugars. To date, many of these studies have focused on the effects of these sugars on hepatic and systemic dyslipidemia and glucose tolerance^1–4^. High sugar feeding has been shown to promote hepatic de novo lipogenesis^5–7^, with both fructose and glucose components contributing to fatty acid and glycerol synthesis^8^. Several studies have also reported decreased glucose tolerance in rodents fed diets high in sucrose^1,3,4^ with alterations in hepatic glucose metabolism considered to have greater roles in this pathology compared to systemic glucose clearance, at least in the early stages. The conversion of dietary glucose to glycogen in liver by the direct pathway is an important component in the maintenance of glucose tolerance. Direct pathway activity is highly dependent on insulin actions, with reduced fluxes under both insulin-deficient and insulin-resistant states^9,10^. In the setting of high sucrose or HFCS feeding, the effects of the fructose component on hepatic glycogen synthesis rates and on the conversion of dietary glucose to glycogen are unclear. On the one hand, fructose is a potent glycogenic precursor^11,12^ that is converted to glycogen via the indirect pathway, thereby potentially competing with glucose for hepatic glycogen synthesis. On the other hand, fructose potentiates the conversion of glucose to glycogen via glucokinase activation^13–15^. High levels of hepatic glycogen also reduces food intake^16,17^ hence any changes in hepatic glycogen metabolism may influence systemic energy balance. The aim of this study was to compare hepatic glycogen synthesis in mice fed with standard chow to mice where the standard chow was supplemented with a mixture of fructose and glucose in the drinking water corresponding to HFCS-55. Given the  ~ 22% excess of fructose over glucose, coupled with previous studies documenting the efficient recruitment of fructose carbons for glycogen synthesis^11,12^, we hypothesized that the fructose component of HFCS-55 would be more efficiently converted to glycogen compared to the glucose component. By integrating deuterated water (^2^H_2_O) measurements of hepatic glycogen sources under natural feeding conditions^9,11,12,18^ with ^13^C-tracers of the glucose and fructose present in the drinking water (Fig. 1), the specific contributions of the HFCS-55 glucose and fructose components to hepatic glycogen synthesis were determined.

## Methods

### Materials

[U-^13^C]Fructose at 99% enrichment was obtained from Omicron Biochemicals Inc., IN, USA and [U-^13^C]glucose at 99% enrichment was manufactured by Cambridge Isotopes Limited, Cambridge, MA, USA and purchased through Tracertec, Madrid, Spain. ^2^H_2_O at 99.8% isotopic enrichment was also manufactured by Cambridge Isotopes Limited and purchased through Tracertec, Madrid, Spain.

### Animal studies

Animal studies were approved by the University of Coimbra Ethics Committee on Animal Studies (ORBEA) and the Portuguese National Authority for Animal Health (DGAV), approval code 0421/000/000/2013. In addition, all animal procedures hereby described were performed in full accordance with DGAV guidelines and European regulations (European Union Directive 2010/63/EU). Twenty one adult male C57BL/6J mice obtained from Charles River Labs, Barcelona, Spain, were housed at the University of Coimbra UC Biotech Bioterium. They were maintained in a well-ventilated environment and a 12 h light/12 h dark cycle. Upon delivery to the Bioterium, mice were provided a 2 week interval for acclimation, with free access to water and standard chow, comprising of 54% mixed carbohydrate, 19% protein and 3% lipid by weight. After this period, twelve of the mice were provided with drinking water solution containing a 55/45 mixture of fructose and glucose. This mixture was present at a concentration of 30% w/v and was provided for a period of 18 weeks. The remaining nine mice were maintained on a standard chow diet for the same period. At the beginning of the final evening, all mice were administered with an intraperitoneal loading dose of 99% ^2^H_2_O containing 0.9 mg/ml NaCl (4 ml/100 g body weight) and the drinking water was enriched to 5% with ^2^H_2_O. For the 12 mice that were provided with the fructose/glucose mixture in their drinking water, this was replaced with mixtures of identical composition but with 20% enriched [U-^13^C]fructose for six of the animals and 20% enriched [U-^13^C]glucose for the remaining six mice. At the end of this dark cycle, mice were deeply anesthetized with ketamine/xylazine and sacrificed by cardiac puncture. Arterial blood was immediately centrifuged and plasma was isolated and stored at − 80 °C. Livers were freeze-clamped and stored at − 80 °C until further analysis.

### Liver glucose and glycogen extraction and monoacetone glucose synthesis

Livers were ground under liquid N_2_ and extracted with methyl *tert*-butyl ether as previously described^8^. For analysis of liver glucose, the aqueous phase was neutralized, lyophilized and analyzed directly by ^13^C NMR. Glycogen from the insoluble pellet was extracted with 30% KOH (2 ml/g of liver) at 70 °C for 30 min. The mixture was then treated with 6% Na_2_SO_4_ (1 ml/g of liver) and glycogen was precipitated with ethanol (7 ml/g of liver). After centrifugation, the solid residue was dried and resuspended in acetate buffer (50 mM, pH 4.5). 20 units of amyloglucosidase from *Aspergillus niger* (Glucose-free preparation, Sigma-Aldrich, Germany) was added and the solution incubated overnight at 55 °C. The supernatant was lyophilized and mixed with 5 ml ^2^H-enriched acetone prepared as described^19^ and 4% sulphuric acid enriched to 2% with ^2^H_2_SO_4_ (v/v). The mixture was stirred overnight at room temperature. The reaction was quenched with water (5 ml, enriched to 2% with ^2^H_2_O), the pH adjusted with HCl (pH 2.0) and the mixture incubated at 40 °C for 5 h. The solution pH was adjusted to 8 with NaHCO_3_ and the samples evaporated to dryness. Monoacetone glucose (MAG) in the residue was extracted with boiling ethyl acetate. Ethyl acetate was evaporated, the residue dissolved in H_2_O and purified by solid phase Discovery DSC-18 3 ml/500 mg disposable columns (Sigma-Aldrich) as previously described^20^.

### NMR analysis

Proton-decoupled ^2^H-NMR spectra of MAG samples were obtained with a Bruker Avance III HD 500 spectrometer using a ^2^H-selective 5 mm probe incorporating a ^19^F-lock channel. Samples were resuspended in 0.5 ml 90% acetonitrile/10% ^2^H-depleted water to which 50 μl of hexafluorobenzene were added. ^2^H-NMR spectra were obtained with a 90° pulse angle, 1.6 s of acquisition time and a 0.1 s interpulse delay. The number of fee-induction decays (f.i.d.) collected ranged from 2,000 to 10,000. Positional ^2^H-enrichments were determined using the MAG methyl signals as an intramolecular standard^19^. To quantify plasma body water ^2^H-enrichments, triplicate 10 µl samples of plasma were analyzed by ^2^H NMR as previously described^21^ but with 50 μl of hexafluorobenzene added to the NMR sample. Proton-decoupled ^13^C NMR spectra were obtained with a Varian VNMRS 600 MHz NMR (Agilent) spectrometer equipped with a 3-mm broadband probe. ^13^C NMR spectra were acquired at 25 °C using a 60° pulse, 30.5 kHz spectral width and 4.1 s of recycling time (4.0 s of acquisition time and 0.1 s pulse delay). The number of acquisitions ranged from 2,000 to 18,000. The summed f.i.d. was processed with 0.2 Hz line-broadening and zero-filled to 512 K before Fourier transform.

^13^C- and ^2^H-NMR spectra were analyzed with ACD/NMR Processor Academic Edition software (ACD/Labs, Advanced Chemistry Development, Inc.).

### Quantifying the contributions of direct pathway, Krebs cycle and triose-P sources to hepatic glycogen from analysis of glycogen ^2^H-enrichment from ^2^H_2_O

The fraction of newly synthesized glycogen and the contributions of direct pathway, indirect-Krebs cycle and indirect-triose-P sources were quantified from enrichment of glycogen positions 2, 5, 6_*S*_ and that of body water as previously described^11,12^. Enrichment of glycogen position 2 was corrected for incomplete exchange of body water and position 2 hydrogens by multiplication with 1.57^11^. Since direct pathway and glycogen-glucose-6-P cycling fluxes both contribute to position 2 enrichment^22^, we reported this activity as direct pathway + glycogen cycling.

The fraction of newly-synthesized glycogen (f_*GLY*_) was calculated as follows:

$${\text{f}}_{GLY} = {1}00 \times\,{^{{2}}} {\text{H2}}_{CORR} /{\text{BW}}$$

where ^2^H2_*CORR*_ is the corrected position 2 enrichment and BW is the body water enrichment. The balance represents the fraction of pre-existing glycogen that did undergo cycling:

1 $${\text{Fraction}}\;{\text{of}}\;{\text{pre-existing}},\;{\text{non-cycled}}\;{\text{glycogen}} = {1}00 - {\text{f}}_{GLY}$$

The contribution of direct pathway + glycogen cycling to newly synthesized glycogen was calculated as follows:

2 $${\text{Direct}}\;{\text{pathway}} + {\text{glycogen}}\;{\text{cycling}}\;\left( \% \right) = {1}00 \times \left( {{1} -\,{^{{2}}}{\text{H5}}/{^{{2}}} {\text{H2}}_{CORR} } \right)$$

where ^2^H5/^2^H2_*CORR*_ is the ratio of position 5 to the corrected position 2 enrichment.

The contribution of the indirect pathway from all sources to newly synthesized glycogen was calculated as follows:

$${\text{Indirect}}\;{\text{pathway}},\;{\text{all}}\;{\text{sources}}\;\left( \% \right) = {1}00 \times\,{^{{2}}} {\text{H5}}/{^{{2}}} {\text{H2}}_{CORR}$$

The contribution of indirect pathway Krebs cycle sources (Indirect_*KC*_) to newly synthesized glycogen was calculated as follows:

3 $${\text{Indirect}}_{KC} \;\left( \% \right) = 100 \times \,{^{2}}H6_{S} /{^{2}} {\text{H}}2_{CORR}$$

where ^2^H6_*S*_ /^2^H2_*CORR*_ is the ratio of position 6_*S*_ to the corrected position 2 enrichment.

The contribution of indirect pathway triose-P sources (Indirect_*TP*_) to newly synthesized glycogen was calculated as follows:

4 $${\text{Indirect}}_{TP} \left( \% \right) = 100 \times \left( {^{2} {\text{H}}5 - \,{^{2}} H6_{S} } \right)/{^{2}} {\text{H}}2_{CORR}$$

### Quantifying the contribution of exogenous [U-^13^C]glucose and direct pathway to hepatic glycogen synthesis

[U-^13^C]glucose is metabolized to [U-^13^C]glycogen via the direct pathway. The fractional contribution of exogenous [U-^13^C]glucose to glycogen synthesis via the direct pathway (Exo-glucose_*DIR*_) was estimated from the ratio of [U-^13^C]glycogen to exogenous [U-^13^C]glucose enrichment as previously described ^20^.

5 $${\text{Exo-glucose}}_{DIR} \;(\%) = 100 \times \left( {\left[{{\text{U-}}{^{13}}{\text{C}}} \right]{\text{glycogen}} \times 5}\right)/f_{GLY}$$

where the factor of 5 accounts for the 20% enrichment of exogenous glucose with [U-^13^C]glucose.

The fraction of glycogen derived from glucose metabolized by the indirect pathway, i.e. via the Krebs cycle (Exo-glucose_*KC*_) was calculated as follows:

6 $${\text{Exo-glucose}}_{KC} \;\left( \% \right) = (100 \times \left( {\left[ {5,6{\text{-}}{^{13}}{\text{C}}_{2} } \right]{\text{glycogen}} \times 1.67} \right) \times 1.5 \times 5)/{\text{f}}_{GLY}$$

where the factor of 1.67 accounts for the formation of 0.67 [4,5,6-^13^C_3_]glycogen_*KC*_ for every [5,6-^13^C_2_]glycogen due to pyruvate cycling; the factor of 1.5 accounts for dilution of both isotopomers by exchange at the level of the Krebs cycle^20^ and the factor of 5 accounts for the [U-^13^C]glucose load being 20% enriched.

### Quantifying the contribution of exogenous [U-^13^C]fructose to hepatic glycogen synthesis

The contribution of [U-^13^C]fructose to glycogen synthesis via Triose-P and Krebs cycle routes were estimated from the enrichment levels of [4,5,6-^13^C_3_]glycogen and [5,6-^13^C_2_]glycogen, respectively. These values were obtained from the carbon 5 multiplet components of the MAG ^13^C NMR spectra as follows:

$$\begin{aligned} & \left[ {4,5,6{\text{-}}{^{13}}{\text{C}}_{3} } \right]{\text{glycogen}}\;\left( \% \right) = {\text{C}}5{\text{Q}}/{\text{C}}5{\text{S}} \times 1.11 \\ & \left[ {5,6{\text{-}}{^{13}}{\text{C}}_{2} } \right]{\text{glycogen}}\;\left( \% \right) = {\text{C}}5{\text{D}}56/{\text{C}}5{\text{S}} \times 1.11 \\ \end{aligned}$$

where C5Q/C5S is the area of the quartet component relative to that of the singlet signal in the MAG carbon 5 multiplet; C5D56/C5S is the area of the doublet 56 component relative to that of the singlet signal in the same multiplet and 1.11 represents the natural abundance ^13^C level.

The contribution of exogenous fructose to glycogen synthesis via the triose-P pathway was determined from the enrichment of [4,5,6-^13^C_3_]glycogen after correction for [4,5,6-^13^C_3_]glycogen that was formed via the Krebs cycle.

$$\left[ {4,5,6{\text{-}}{^{13}}{\text{C}}_{3} } \right]{\text{glycogen}}_{TP} = \left[ {4,5,6{\text{-}}{^{13}}{\text{C}}_{3} } \right]{\text{glycogen}}_{total} - \left[ {4,5,6{\text{-}}{^{13}}{\text{C}}_{3} } \right]{\text{glycogen}}_{KC}$$

where [4,5,6-^13^C_3_]glycogen_*TP*_ is the estimated enrichment of [4,5,6-^13^C_3_]glycogen derived from triose-P, [4,5,6-^13^C_3_]glycogen_*total*_ is the measured [4,5,6-^13^C_3_]glycogen enrichment, and [4,5,6-^13^C_3_]glycogen_*KC*_ is the estimated enrichment of [4,5,6-^13^C_3_]glycogen derived via the Krebs cycle. This was calculated from the following equation:

$$\left[ {4,5,6{\text{-}}{^{13}}{\text{C}}_{3} } \right]{\text{glycogen}}_{KC} = \left[ {5,6{\text{-}}{^{13}}{\text{C}}_{2} } \right]{\text{glycogen}} \times 0.67$$

where the factor of 0.67 accounts for the lower yield of [4,5,6-^13^C_3_]glycogen_*KC*_ relative to [5,6-^13^C_2_]glycogen due to pyruvate cycling^23^.

The fractional contribution of fructose to newly-synthesized glycogen via triose-P (Exo-fructose_*TP*_) was calculated as follows:

7 $${\text{Exo-fructose}}_{TP} \;\left( \% \right) =\left (100 \times \left[ {4,5,6{\text{-}}{^{13}}{\text{C}}_{3} } \right]{\text{glycogen}}_{TP} \times 5\right)/{\text{f}}_{GLY}$$

where the factor of 5 accounts for the 20% enrichment of exogenous fructose with [U-^13^C]fructose.

The fraction of glycogen derived from fructose via the Krebs cycle (Exo-fructose_*KC*_) was calculated as follows:

8 $${\text{Exo-fructose}}_{KC} \;\left( \% \right) = ({1}00 \times \left( {\left[ {{5},{6\text{-}}{^{{{13}}}}{\text{C}}_{{2}} } \right]{\text{glycogen}} \times {1}.{67}} \right) \times {1}.{5} \times {5})/{\text{f}}_{GLY}$$

where the factor of 1.67 accounts for the formation of 0.67 [4,5,6-^13^C_3_]glycogen_*KC*_ for every [5,6-^13^C_2_]glycogen due to pyruvate cycling; the factor of 1.5 accounts for dilution of both isotopomers by exchange at the level of the Krebs cycle^20^ and the factor of 5 accounts for the [U-^13^C]fructose load being 20% enriched.

### Statistics

All results are presented as means ± standard error of the mean (S.E.M.) All datasets were submitted to a Shapiro–Wilk normality test and homoscedasticity test (F test of equality of variances). If both groups presented a normal distribution, then an unpaired Student t-test was applied (Welch corrected if variances were unequal). Otherwise, the Mann–Whitney *U* test was employed.

## Results

The mean weight of the mice whose chow was supplemented with fructose and glucose in the drinking water, hereafter referred to as HFCS-55 mice, was not significantly different from the group fed normal chow, hereafter referred to as NC mice (34.0 ± 3.5 g vs 35.7 ± 4.2 g, *p* = 0.34). While the bolus of ^2^H_2_O was adjusted to the weight of each animal, there was a significant difference in body water ^2^H enrichment between HFCS-55 and NC mice (Table 1). This likely reflects differences in body composition between the two groups, with the HFCS-55 mice having a higher fraction of adipose tissue, hence a lower fraction of water. Hepatic glycogen concentrations tended to be higher in HFCS-55 compared to NC mice (38 ± 3 vs 30 ± 3 mg/g wet weight of liver, *p* = 0.07).

**Table 1:** Summary of liver glycogen positional ^2^H-enrichments (H1–H6) and body water ^2^H-enrichments (BW) for mice administered with ^2^H_2_O during feeding with normal chow (NC), normal chow plus HFCS-55 in the drinking water with enriched with [U-^13^C]glucose (HFCS-55 [U-^13^C]Glu) and normal chow plus HFCS-55 in the drinking water with enriched with [U-^13^C]fructose (HFCS-55 [U-^13^C]Fru).

Diet	Summary of glycogen positional and body water (BW) ^2^H-enrichments									
	H1	H2	H2_corr_	H3	H4	H5	H6R	H6S	BW	H2_corr_/BW
NC (n = 9)	1.56 ± 0.11	3.28 ± 0.21	5.14 ± 0.33	1.26 ± 0.08	1.65 ± 0.10	1.74 ± 0.12	1.15 ± 0.09	1.20 ± 0.09	4.46 ± 0.25	1.14 ± 0.10
HFCS-55 [U^13^C]Glu (n = 6)	0.90 ± 0.06	2.98 ± 0.22	4.69 ± 0.34	1.42 ± 0.17	1.68 ± 0.19	1.80 ± 0.22	0.60 ± 0.07	0.60 ± 0.06	6.01 ± 0.15	0.78 ± 0.06
HFCS-55 [U^13^C]Fru (n = 6)	0.74 ± 0.10	2.71 ± 0.27	4.25 ± 0.42	1.12 ± 0.13	1.43 ± 0.16	1.45 ± 0.20	0.56 ± 0.11	0.50 ± 0.10	5.75 ± 0.24	0.73 ± 0.04
HFCS-55 total (n = 12)	0.82 ± 0.06	2.85 ± 0.17	4.47 ± 0.27	1.27 ± 0.11	1.56 ± 0.13	1.63 ± 0.15	0.58 ± 0.06	0.55 ± 0.06	5.88 ± 0.14	0.76 ± 0.04
*p* values (HFCS-55 total *versus* NC)	< **0.001**^T^	0.111^M^	0.111^M^	0.382^M^	0.219^M^	0.219^M^	< **0.001**^M^	< **0.001**^T^	< **0.001**^T^	**0.006**^T^

Each value represents a mean ± S.E.M and statistically significant comparisons are highlighted in bold. H2_*corr*_—Position 2 enrichment after correction for incomplete exchange of ^2^H between water and position 2 of glucose-6-P. ^T^Student’s *T* Test; ^M^Mann–Whitney *U* test.

### Hepatic glycogen enrichment from ^2^H_2_O

**Figure 2:**
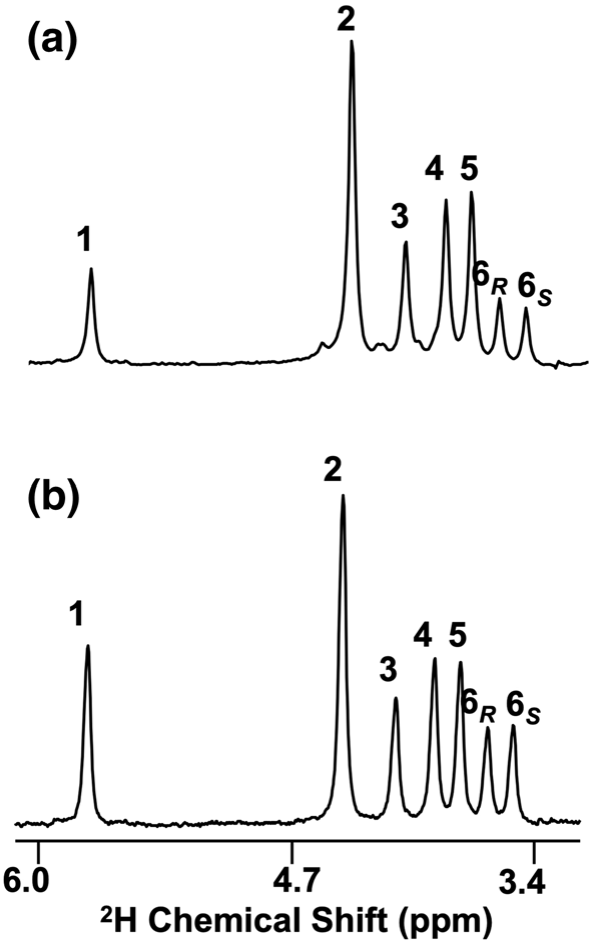
^2^H NMR spectra of monoacetone glucose derived from liver glycogen of a mouse fed standard chow supplemented with a 55/45 mix of 20% enriched [U-^13^C]fructose and unlabeled glucose in the drinking water (**a**) and a mouse fed with standard chow (**b**).

Representative ^2^H NMR spectra of MAG derived from hepatic glycogen of HFCS-55 and NC mice are shown in Fig. 2. The positional ^2^H-enrichments obtained from these spectra, including the position 2 enrichment corrected for incomplete exchange at the level of glucose-6-P (H2_*corr*_) are shown in Table 1. For the control group, H2_*corr*_ was not statistically different from body water enrichment, as seen by H2_*corr*_/BW of ~ 1.0 (see Table 1) indicating that all of the glycogen had been newly synthesized and/or had undergone cycling with glucose-6-P during the interval that ^2^H_2_O was administered. For the HFCS-55 animals, H2_*corr*_ was significantly less than BW indicating that only about 75% of the hepatic glycogen had been newly synthesized over the same period. For both NC and HFCS-55 mice, about two-thirds of the newly-formed glycogen was accounted by direct pathway + cycling, with the indirect pathway contributing about one-third. As previously demonstrated from the analysis of position 5 and 6_*S*_ enrichments, indirect pathway fluxes can be resolved into contributions from substrates that directly feed the triose-P pool, such as fructose and glycerol, and contributions from precursors such as alanine and pyruvate that are converted to triose-P via the Krebs cycle^11,12^. As is apparent in the ^2^H NMR spectra of Fig. 2, the difference in signal intensities between ^2^H5 and ^2^H6_S_ is larger for HFCS-55 compared to NC. This translates to a higher contribution of Triose-P sources to indirect pathway fluxes relative to Krebs cycle precursors for HFCS-55 mice and is consistent with the utilization of fructose as a glycogenic precursor for these animals.

### ^13^C-isotopomer analysis of hepatic metabolite enrichment from exogenous [U-^13^C]glucose and [U-^13^C]fructose

The aqueous fraction of the liver tissue was also analyzed by ^13^C NMR in order to determine the enrichment of glucose, fructose and other metabolites immediately prior to their incorporation into hepatic intermediary metabolism. Representative spectra are shown in Figure 1 of the supplementary data. The most abundant ^13^C-enriched metabolite observed in these fractions was glucose, with lesser amounts of lactate and barely detectable levels of alanine, glutamate and other amino acids. For the mice administered with exogenous [U-^13^C]glucose, ^13^C-multiplet signals representing [U-^13^C]glucose were the dominant features consistent with absorption and transport of exogenous glucose to the liver. In comparison, signals from partially-enriched glucose ^13^C-isotopomers generated via Cori cycle cycling and/or pentose phosphate pathway fluxes were relatively minor. From analysis of the [U-^13^C]glucose isotopomer components of these spectra, enrichment of [U-^13^C]glucose in liver tissue was estimated to be 4.6% (Table 2). This represents an approximately four-fold dilution in enrichment when compared to the exogenous 20% [U-^13^C]glucose precursor. Remarkably, for the mice provided with [U-^13^C]fructose, there was no detectable fructose in the hepatic aqueous fractions from any of the animals, as seen by a complete lack of resolvable ^13^C signals resonating at 104.2 and 100.8 ppm, representing the fructose carbons 1α and 1β, respectively. However, the glucose present in these fractions showed strong enrichment from ^13^C-isotopomers such as [1,2,3-^13^C_3_]- and [4,5,6-^13^C_3_]glucose—the expected products of fructose metabolism to triose-P followed by gluconeogenesis to form glucose. ^13^C-Lactate and alanine isotopomers were also observed in similar amounts to that seen in mice given [U-^13^C]glucose.

**Table 2:** Liver glucose and glycogen ^13^C-isotopomer enrichments from six mice fed normal chow plus HFCS-55 in the drinking water enriched with [U-^13^C]glucose (HFCS-55 [U-^13^C]Glu), and six mice fed normal chow plus HFCS-55 in the drinking water with enriched with [U-^13^C]fructose (HFCS-55 [U-^13^C]Fru).

Group	Liver glucose ^13^C-isotopomers (%)	Liver glycogen ^13^C-isotopomers (%)			
	[U-^13^C]glucose	[U-^13^C] glycogen	[4,5,6-^13^C_3_] glycogen	[5,6-^13^C_2_] glycogen	[4,5-^13^C_2_] glycogen
HFCS-55 [U-^13^C]Glu (n = 6)	4.58 ± 0.61	2.57 ± 0.41	n.d	0.04 ± 0.02	0.03 ± 0.02
HFCS-55 [U-^13^C]Fru (n = 6)	n.d	0.55 ± 0.06	2.59 ± 0.14	0.10 ± 0.01	0.10 ± 0.01

*n.d.* not determined.

**Figure 3:**
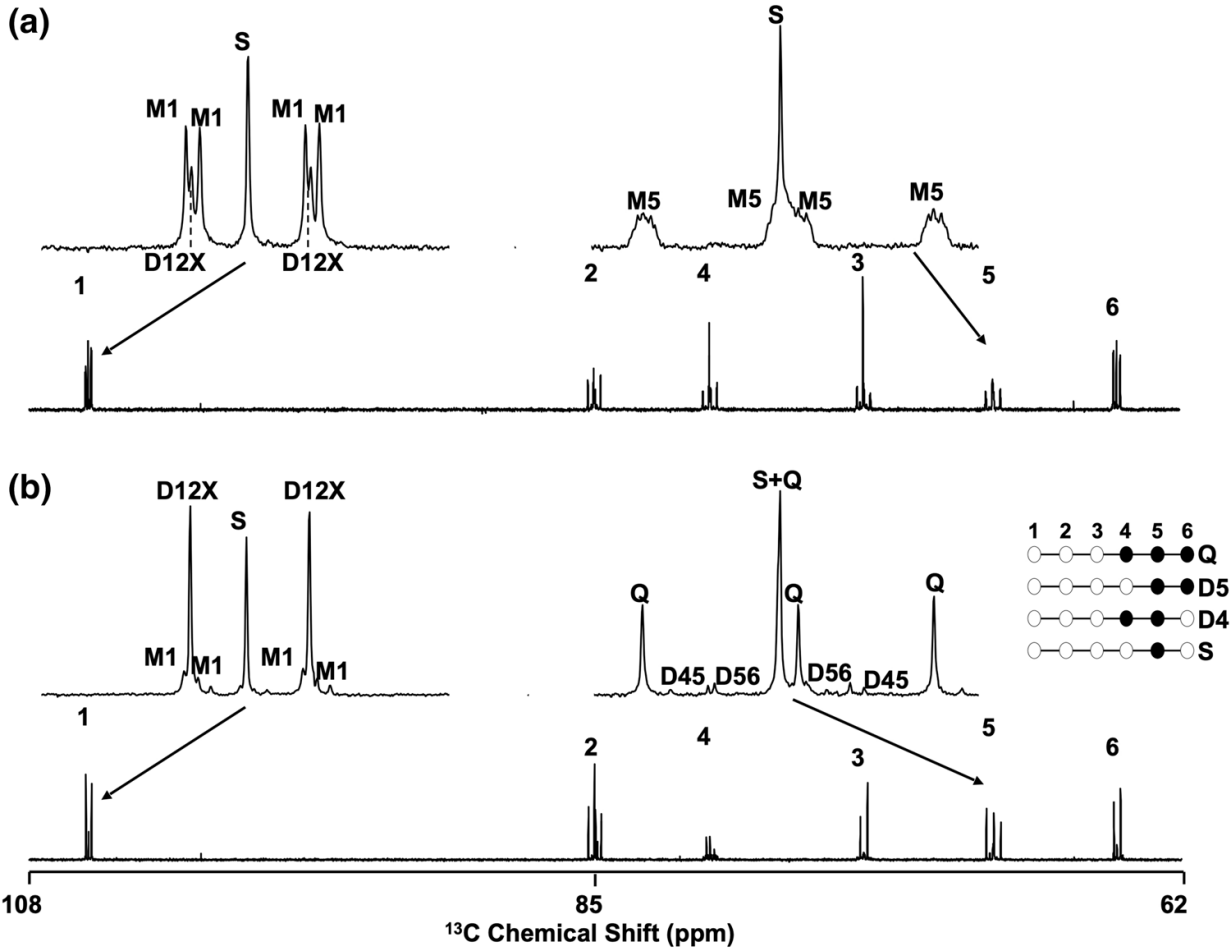
^13^C NMR spectra of hepatic glycogen following derivatization to monoacetone glucose obtained from a mouse fed a glucose/fructose mixture enriched with [U-^13^C]glucose (**a**) and [U-^13^C]fructose (**b**). For the ^13^C NMR multiplets, **S** = carbon singlet resonance; **D12X** = carbon 1 doublet from ^13^C–^13^C-coupling between carbon 1 and carbon 2 and ± ^13^C in carbon 3; **M1** = carbon 1 multiplet from ^13^C–^13^C-couplings between carbon 1, carbon 2 and carbon 5; **M5** = carbon 5 multiplet from ^13^C–^13^C-couplings between carbon 5, carbon 6, carbon 3 and carbon 1; **D56** = carbon 5 doublet from ^13^C–^13^C-coupling between carbon 5 and a neighboring ^13^C in position 6; **D45** = carbon 5 doublet from ^13^C–^13^C-coupling between carbon 5 and a neighboring ^13^C in position 4; **Q** = carbon 5 quartet from ^13^C–^13^C-coupling between carbon 5 and neighboring ^13^C in both positions 4 and 6. To the right of the carbon 5 multiplet of spectrum (**b**) are shown the ^13^C-isotopomers represented by the **S**, **D45**, **D56** and **Q** components.

^13^C NMR spectra of hepatic glycogen obtained from mice provided with [U-^13^C]glucose and [U-^13^C]fructose tracers are shown in Fig. 3. For mice that received [U-^13^C]glucose (Fig. 3a), the spectra were dominated by complex multiplets representing [U-^13^C]glucosyl units that originated via direct pathway metabolism of hepatic glucose. ^13^C-multiplet signals representing Cori cycling or indirect pathway metabolism of [U-^13^C]glucose, such as the D45 and D56 components of the carbon 5 multiplet, were either not observed or barely detectable. In comparison, ^13^C multiplet signals representing [1,2-^13^C_2_]- and/or [1,2,3-^13^C_3_]glucose such as the D12X, were more abundant, consistent with pentose phosphate pathway activity^24^. For the mice that received [U-^13^C]fructose, incorporation of exogenous fructose into glycogen was confirmed by the appearance of glycogen carbon 5 multiplet signals in the ^13^C NMR spectrum. These correspond to glycogen ^13^C-isotopomers from the glycogenic metabolism of [U-^13^C]fructose (see Figs. 1 and 3b). The carbon 5 quartet (C5Q) representing [4,5,6-^13^C_3_]glycogen was the dominant component, accounting for  ~ 95% of the total multiplet signals. In comparison, the D45 and D56 components representing passage of the ^13^C-label through the Krebs cycle, were minor signals. This indicates that the principal route of fructose conversion to glycogen was predominantly via triose-P and hexose-P intermediates. In the carbon 1 resonance, there was a minor contribution from the M1 multiplet. While [U-^13^C]fructose is predicted to generate discrete ^13^C-isotopomers for each of the triose halves of glucose-6-P, a small proportion of glucose-6-P will be synthesized with ^13^C in both triose moieties thereby accounting for the M1 signal. Consistent with this, a close inspection of the carbon 5 Q signals revealed some residual clusters of M5 at their base.

### Estimates of exogenous glucose, fructose and other substrate contributions to hepatic glycogen synthesis

**Figure 4:**
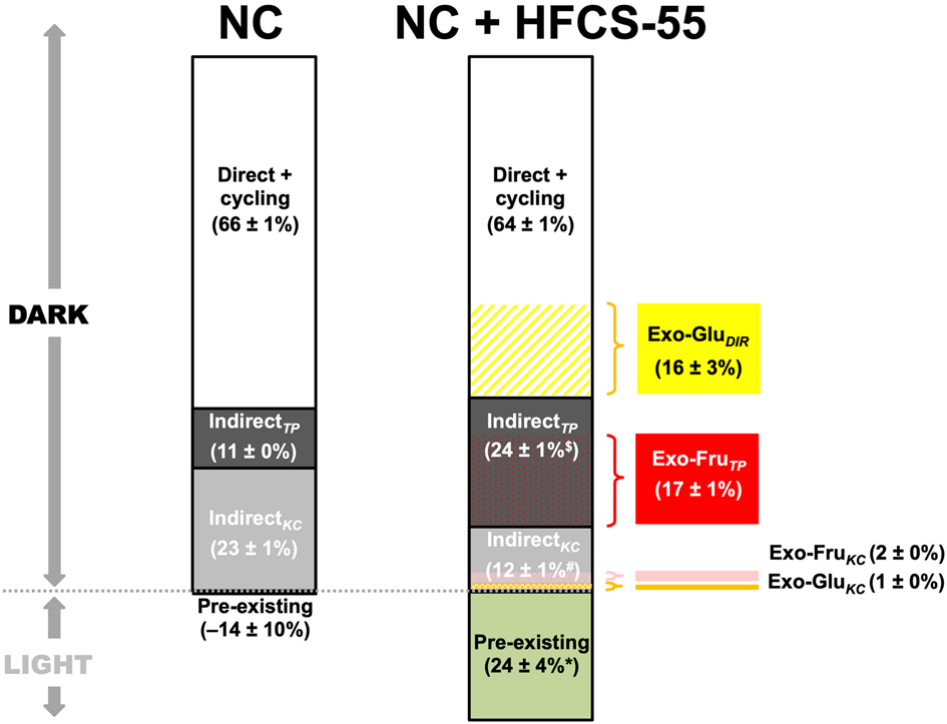
Sources of hepatic glycogen synthesis in mice fed normal chow (NC) and mice fed normal chow plus a mixture of fructose and glucose in the drinking water (HS). Pre-existing glycogen was assumed to have been present at the end of the light phase of the previous day while all newly-synthesized glycogen, arbitrarily set to 100%, was defined as having been formed entirely during the following dark period. For NC mice these include the fraction of pre-existing glycogen that had not undergone cycling with glucose-6-P (Eq. 1); direct pathway + cycling (Eq. 2), indirect pathway via the Krebs cycle (Indirect_*KC*_, Eq. 3); and indirect pathway via triose-P (Indirect_*TP*_, Eq. 4). For the HS mice, these parameters are accompanied by direct pathway contribution of exogenous glucose shown in yellow (Exo-Glu_*DIR*_, Eq. 5), indirect pathway Krebs cycle contribution of exogenous glucose shown in orange (Exo-Glu_*KC*_, Eq. 6), exogenous fructose contribution via Triose-P shown in red (Exo-Fru_*TP*_, Eq. 7) and exogenous fructose contribution via the Krebs cycle shown in pink (Exo-Fru_*KC*_, Eq. 8). *p < 0.005 versus NC, ^**#**^*p* < 10^–5^ versus NC, ^**$**^*p* < 10^–7^ versus NC.

The contributions of exogenous and endogenous sources to hepatic glycogen synthesis during a single night for NC and HFCS-55 mice are summarized in Fig. 4. For NC mice, estimates of pre-existing glycogen (− 14 ± 10%) indicate that glycogen turnover, defined as net synthesis and/or cycling with glucose-6-P, was fully complete over the dark cycle. Two-thirds of the glycogen was accounted by direct pathway + cycling and one-third was synthesized from indirect pathway sources, with the Krebs cycle providing the majority of indirect pathway carbons. For HFCS-55 mice, pre-existing non-cycled glucosyl units accounted for a significant fraction of the total liver glycogen. Of the newly-synthesized fraction, direct pathway plus cycling accounted for about two-thirds with one-third originating from indirect pathway sources. However, in contrast to the NC mice, triose-P substrates were the dominant source of indirect pathway carbons, supplying twice that of Krebs cycle sources. This profile is consistent with the contributions of exogenous glucose and fructose derived from the ^13^C-isotopomer analysis. The majority of indirect pathway triose-P phosphate sources can be attributed to exogenous fructose, confirming our previous observations of its effects on indirect pathway fluxes in both mice and rats^11,12^.

## Discussion

In this study, the effects of supplementing a normal chow diet with a fructose and glucose formulation corresponding to HFCS-55 on hepatic glycogen synthesis was studied in mice during ad-libitum feeding. In addition to comparing excursions in glycogen levels between the start and end of the night and determining the sources of glycogen synthesis in HFCS-55 mice relative to NC, individual contributions of the fructose and glucose components to glycogen synthesis were also measured for the HFCS-55 mice. Although the high-sugar feeding protocol used in our study is similar to that described in other reports of rodent models, there are important caveats and limitations in applying the physiological and biochemical information gained from such studies to better understand the effects of excessive sugar intake in humans. Perhaps the largest discrepancy between our rodent model and humans that are categorized as high sugar consumers is that the sugar load, its caloric contribution to total energy intake, and the extent of caloric excess relative to the control diet, is almost certainly far greater for the mouse compared to the human. Therefore, it is possible that some of our metabolic observations may reflect excessive caloric intake rather than the effects of the individual sugars per se^25^. Our methodology can nevertheless be translated to humans by integrating ^2^H_2_O and ^13^C-sugar administration with a noninvasive “chemical biopsy” of hepatic UDP-glucose ^2^H and ^13^C enrichments by ingestion of Paracetamol and analysis of urinary Paracetamol glucuronide^26^.

At the start of the dark cycle, there was a substantial amount of pre-existing, non-cycled glycogen in HFCS-55 while estimates for NC yielded a nominal negative value. Longitudinal studies of hepatic glycogen excursions measured biochemically in mice fed normal chow over a 12 h/12 h light and dark cycle indicate that glycogen stores can drop to 10–15% of their maximal post-absorptive values^27,28^. As shown by Eq. (1) of the methods section, our estimate of the pre-existing glycogen fraction is dependent on the value of position 2 enrichment after correction for incomplete exchange of body water and position 2 hydrogens (^2^H2_*corr*_). Given that the correction factor was derived from mice fed fructose/glucose loads that resembled those given to HFCS-55 mice^11^, it is possible that it understates the extent of H2-body water exchange in mice fed normal chow, thereby resulting in a systematic underestimate of pre-existing glycogen in NC mice.

Given that HFCS-55 mice ingested a significant amount of glucose and fructose every time they drank water during daylight hours, this means that their baseline glycogen levels were likely higher compared to NC mice. On average, HFCS-55 mice had 21% more total glycogen than NC at the end of the night but 27% of this was pre-existing. Therefore, net glycogen synthesis during the night was no higher in HFCS-55 compared to NC mice.

While total indirect pathway contributions were very similar for HFCS-55 and NC (36% and 34%, respectively), triose-P was the dominant source for HFCS-55 mice while the Krebs cycle was the principal source for NC mice. In a previous study of rats that were provided with 35% sucrose w/v in their drinking water in the same experimental setting as this study, a similar shift in indirect pathway sources was observed but there was also an overall increase in the indirect pathway contribution^12^. However, since this earlier study did not correct for incomplete exchange of the glucose-6-P position 2 hydrogen with body water, the total indirect pathway contributions could have been overestimated. However, since the proportion of Krebs cycle and Triose-P sources contributing to the indirect pathway is not influenced by position 2 enrichment, these parameters can be compared between present and previous studies. In the present study, we verified with ^13^C-tracers that the increased triose-P contributions to indirect pathway flux were indeed explained by the glycogenic metabolism of fructose.

In the HFCS-55 mice, the ^13^C-isotopomer data also provided new insights on how exogenous glucose was converted to glycogen in the presence of fructose. The direct pathway, which requires glucokinase activation, accounted for essentially for all of this process with only residual contributions via the indirect pathway. The role of fructose in this process in the context of high sugar feeding is ambiguous. On the one hand, fructose potentiates the activation of glucokinase via its fructose-1-phosphate metabolite^29–31^, but this can be effected by very low levels of dietary fructose^14,15,31^. It also contributes to the activation of ChREBP^32^, a transcription factor that promotes the transcription of glycolytic pathway enzymes and acts synergistically with glucokinase to potentiate glucose phosphorylation to glucose-6-phosphate^33^. On the other hand, high levels of dietary fructose and glucose promote hepatic insulin resistance^34–36^, which among other things, would be expected to decrease insulin-mediated activation of glucokinase^37^. Given this possibility, coupled with the excess of fructose over glucose in the HFCS-55 formulation, we hypothesized that the fructose component would contribute a significantly higher fraction of glycogen synthesis compared to glucose. Since fructose is exclusively metabolized to glycogen via the indirect pathway, thereby bypassing glucokinase, we also anticipated a significantly higher indirect pathway contribution for HFCS-55 compared to NC mice. Our results do not support either prediction. The total contributions from HFCS-55 fructose and glucose components were similar (16% and 17%, respectively), while total indirect pathway contributions were 34% and 36% for NC and HFCS-55 mice, respectively.

The glucose component of HFCS-55 accounted for about one-quarter of the glycogen that was synthesized via the direct pathway and/or participated in cycling with glucose-6-P. A substantial direct pathway contribution would be expected from the chow per se, which was 54% by weight of carbohydrate (43% from corn starch, 7% from maltodextrin and 4% from sucrose), representing 60% of total calories. Analysis of liver glucose from HS mice showed a ~ fivefold dilution of the 20% enriched [U-^13^C]glucose in the drinking water by endogenous glucose sources. Assuming that these sources were entirely derived from absorbed glucose, an estimate of direct pathway contributions can be made from the ratio of [U-^13^C]glycogen/liver [U-^13^C]glucose enrichments. This estimate (75 ± 7%) exceeds the 64% obtained from the ^2^H_2_O method and likely reflects an incomplete suppression of hepatic gluconeogenesis.

In healthy humans, substrate levels of fructose were shown to increase the turnover of UDP-glucose while both glucose disposal and endogenous glucose production rates were unchanged^38^, indicative of increased cycling between hepatic glycogen and glucose-6-P. While resolution of total direct pathway contributions and glycogen-glucose-6-P cycling was not possible in the present study, in principle this could be achieved in our experimental setting by supplementing ^2^H_2_O with a combination of [1-^2^H,1-^13^C]glucose and [2-^2^H,2-^13^C]glucose tracers^39^.

In summary, mice whose normal chow was supplemented with a fructose/glucose formulation corresponding to HFCS-55 in the drinking water had similar overall rates of hepatic glycogen deposition during nocturnal feeding and similar indirect pathway contributions to glycogen synthesis. Despite the excess of exogenous fructose over glucose, both were utilized to a similar extent, with exogenous glucose being incorporated almost exclusively by the direct pathway and fructose mainly through the indirect pathway via triose-P. This flux was offset by a decreased Krebs cycle contribution to the indirect pathway.

## Supplementary information

Supplementary Information.

## Data Availability

The datasets generated during and/or analysed during the current study are available from the corresponding author on reasonable request.
