# Supplementary material for: Determining the contribution of a high-fructose corn syrup formulation to hepatic glycogen synthesis during ad-libitum feeding in mice
Source: Sci Rep. 2020 Jul 30;10:12852. doi: 10.1038/s41598-020-69820-3 (PMC7393509; doi:10.1038/s41598-020-69820-3)
Supplement: Supplementary file 1 — Supplementary Information. [file 41598_2020_69820_MOESM1_ESM.pdf]

Determining the Contribution of a High-Fructose Corn Syrup Formulation to Hepatic Glycogen Synthesis During Ad-libitum Feeding in Mice

Giada Di Nunzio[#], Getachew D. Belew[#], Alejandra N. Torres , Joao Gabriel Silva, Luis P. Silva, Cristina Barosa, Ludgero Tavares, and John G. Jones*

Center for Neurosciences and Cell Biology, University of Coimbra, Portugal

[#]Joint first authors

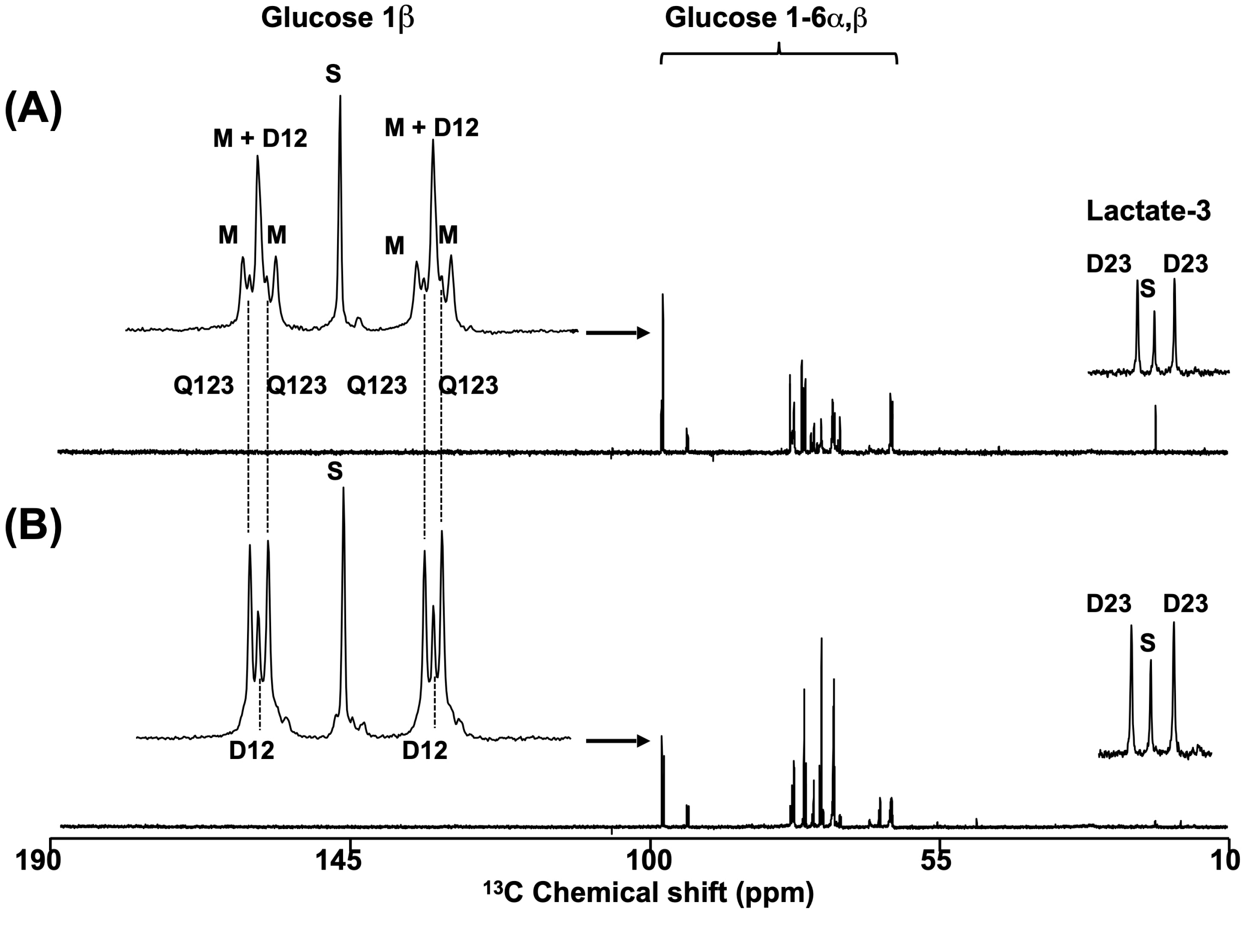

Supplementary Data, Figure 1: ^{13}C NMR spectra of liver aqueous fractions from mice provided with glucose/fructose mixtures enriched with [U-^{13}C]glucose (**A**) and [U-^{13}C]fructose (**B**). The

lactate carbon 3 and glucose carbon 1β signals are shown in expanded form and the multiplet components indicated as follows: **S** = natural-abundance singlet, **D23** = doublet from ^{13}C-^{13}C-coupling between lactate carbon 3 and carbon 2; **M** = multiplet from coupling of glucose carbon 1β with carbons 2β, 3β and 6β and representing [U-^{13}C]glucose; **Q123** = quartet from coupling of glucose carbon 1β with carbons 2β and 3β and representing [1,2,3-$^{13}C_3$]glucose; **D12** = doublet from coupling of glucose carbon 1β with carbon 2β and representing [1,2-$^{13}C_2$]glucose.
